# Multi-omics analyses of early liver injury reveals cell-type-specific transcriptional and epigenomic shift

**DOI:** 10.1186/s12864-021-08173-1

**Published:** 2021-12-18

**Authors:** Maciej Migdał, Eugeniusz Tralle, Karim Abu Nahia, Łukasz Bugajski, Katarzyna Zofia Kędzierska, Filip Garbicz, Katarzyna Piwocka, Cecilia Lanny Winata, Michał Pawlak

**Affiliations:** 1 International Institute of Molecular and Cell Biology in Warsaw, Laboratory of Zebrafish Developmental Genomics, 4 Ks. Trojdena Street, 02-109 Warsaw, Poland; 2 Nencki Institute of Experimental Biology, Laboratory of Cytometry, Warsaw, Poland; 3 Department of Experimental Hematology, Institute of Hematology and Transfusion Medicine, ul. Indiry Gandhi 14, 02-776 Warsaw, Poland; 4 Department of Oncologic Pathology, Dana-Farber Cancer Institute, Harvard Medical School, Boston, USA

**Keywords:** Liver, Hepatocytes, Stellate cells, Endothelial cells, Chromatin, Transcriptomics, ATAC-seq, RNA-seq, Genomics, Epigenomics, Zebrafish

## Abstract

**Background:**

Liver fibrosis is a wound-healing response to tissue injury and inflammation hallmarked by the extracellular matrix (ECM) protein deposition in the liver parenchyma and tissue remodelling. Different cell types of the liver are known to play distinct roles in liver injury response. Hepatocytes and liver endothelial cells receive molecular signals indicating tissue injury and activate hepatic stellate cells which produce ECM proteins upon their activation. Despite the growing knowledge on the molecular mechanism underlying hepatic fibrosis in general, the cell-type-specific gene regulatory network associated with the initial response to hepatotoxic injury is still poorly characterized.

**Results:**

In this study, we used thioacetamide (TAA) to induce hepatic injury in adult zebrafish. We isolated three major liver cell types - hepatocytes, endothelial cells and hepatic stellate cells - and identified cell-type-specific chromatin accessibility and transcriptional changes in an early stage of liver injury. We found that TAA induced transcriptional shifts in all three cell types hallmarked by significant alterations in the expression of genes related to fatty acid and carbohydrate metabolism, as well as immune response-associated and vascular-specific genes. Interestingly, liver endothelial cells exhibit the most pronounced response to liver injury at the transcriptome and chromatin level, hallmarked by the loss of their angiogenic phenotype.

**Conclusion:**

Our results uncovered cell-type-specific transcriptome and epigenome responses to early stage liver injury, which provide valuable insights into understanding the molecular mechanism implicated in the early response of the liver to pro-fibrotic signals.

**Supplementary Information:**

The online version contains supplementary material available at 10.1186/s12864-021-08173-1.

## Background

Liver injury is a rising public health concern, especially in European and North American countries. Its increasing prevalence leads to an expanding body of work regarding the molecular mechanisms present in advanced liver disease, however our knowledge about the earliest stages of liver injury is still limited. Liver injury is manifested by the formation of fibrous tissue as a result of ECM deposition at the site of injury [1]. Progressive fibrous scar formation may distort normal liver structure by formation of septa and nodules of regenerating hepatocytes (HEPs) leading to impaired portal blood flow and formation of cirrhotic architecture [2]. Liver cirrhosis is the end-stage of hepatic fibrosis affecting about 0.1% of the European population [1]. The most serious outcome of cirrhosis is hepatocellular carcinoma (HCC), constituting 70-90% of cases of primary liver cancer [1]. The predominant causes of liver fibrosis are chronic excessive alcohol consumption, viral hepatitis B and C and non-alcoholic fatty liver disease (NAFLD), the latter becoming a major concern with the increasing incidence of obesity in Europe and the USA [1].

Liver parenchymal cells, HEPs, are the most abundant cell subpopulation in this organ in mammals, constituting ca. 85% of the total liver cell mass [3]. Under physiological conditions, HEPs are responsible for a wide range of functions, including carbohydrate, fatty acid and protein metabolism as well as immune response [3]. Upon liver damage, HEPs are a source of reactive oxygen species, pro-inflammatory signals as well as cytokines, taking part in the activation of repair pathways [3].

Hepatic stellate cells (HSCs) comprise 8% of the total liver cell population [4]. Under normal physiological conditions, these mesenchymal cells reside in the space of Disse, maintaining a quiescent state, storing vitamin A in cytoplasmic lipid droplets [5]. Upon liver damage, HSCs are activated and transdifferentiate into myofibroblast-like cells. Their activation is triggered by multiple autocrine and paracrine signals, such as transforming growth factor (TGFβ), SMAD3, protein platelet-derived growth factor receptor (PDGF), vascular endothelial growth factor (VEGF) and connective tissue growth factor (CTGF) [6]. In an active state, HSCs are the primary ECM-producing cell population, resulting in the creation of a temporary scar tissue at the damaged site. Active HSCs produce cytokines and growth factors, promoting liver regeneration. In chronic liver disease, however, the reoccurring HSC activation may result in permanent scar formation, resulting in sections of non-functional liver tissue [5].

Endothelial cells in the liver are found mainly lining the inner walls of the sinusoidal blood vessels (liver sinusoidal endothelial cells - LSECs). LSECs are highly specialized, forming a permeable barrier by virtue of their fenestrae, between hepatocyte membranes and blood vessel lumen. The presence of fenestrae, combined with the absence of a basement membrane, contribute to making the LSECs the most endocytosis-capable cell population in the human body [7]. LSECs regulate the tone of hepatic blood vessels and maintain the quiescent state of HSCs [7].

In response to chronic hepatotoxic injury, various molecular and cellular factors interact with HEPs and LSECs, leading to sequential activation of HSCs [8]. This in turn initiates the perpetuation phase, hallmarked by proliferative, contractile and inflammatory phenotype characterized by increased production of ECM proteins including collagens, fibronectin, decorin, elastin and proteoglycans [2, 9]. The understanding of molecular mechanisms of hepatic fibrosis has markedly increased due to the availability of liver fibrosis models such as cell culture systems, rodent model systems and biopsied human material [10]. However, our knowledge of cell-type-specific gene regulatory networks and epigenetic hallmarks associated with the initial response to hepatotoxic injury is still lacking, mainly due to the challenges of studying cell interactions and their behaviour in a living organism. Such knowledge is crucial for accurate diagnosis and development of new therapeutic approaches targeting liver fibrosis and related disorders.

The zebrafish (*Danio rerio*) has emerged as a useful model organism for studying the mechanism of liver disease in vivo, both in larvae and adult individuals [11–13]. Despite the distinct architecture between mammalian and zebrafish liver, they contain similar main cell types, including HEPs, endothelial cells (ECs) and HSCs, with conserved function and gene expression profiles [5, 14, 15]. To dissect the molecular mechanisms regulating the initiation of hepatic fibrosis and understand the interplay between genetic and epigenetic signals in this process, we utilized the model of thioacetamide-induced liver injury in adult zebrafish and characterized cell-type-specific changes at both transcriptome and epigenome level in three main liver cell types. Thioacetamide (TAA) is a potent hepatotoxin that has been widely used to induce acute and chronic liver injury in rodent models [16–18]. There is a wide variation in the administration routes and time of exposure between studies, but most commonly a regimen of intraperitoneal injections of 100-200 mg/kg of body mass 2-3 times per week for over 6 weeks has been used to induce liver fibrosis and cirrhosis [19]. TAA has also been utilized to induce liver injury in zebrafish larvae, establishing it as a model for steatohepatitis [13]. The larvae used in the cited study were exposed to 0.025% TAA for 10 days starting at 72 h post-fertilization (hpf), when the embryonic liver becomes functional. At 5 days post-fertilization the embryos exhibited molecular markers of apoptosis and steatohepatitis, which continued until the end of the treatment. TAA has also been used in juvenile zebrafish, where intraperitoneal injections of 300 mg/kg b.m. three times a week induced steatosis [20].

We employed three transgenic zebrafish lines to isolate the respective cell populations: HEPs (*Tg(fabp10a:dsRed)*), HSCs (*Tg(hand2:EGFP)*), and ECs (*Tg(kdrl:ras-mCherry)*). We implemented a machine learning technique known as self-organizing maps (SOMs) to generate whole genome expression profiles of both physiological state and early response to liver injury from the three studied cell types [21]. The integration of this data with genome-wide open chromatin maps (ATAC-seq) from corresponding samples allowed to uncover specific gene and chromatin signatures of the studied cell populations. Our analysis revealed that early response of the liver to pro-fibrotic signals is manifested in cell-type specific transcriptome and epigenome rearrangements and identified molecular hallmarks of this process. This work provides a step towards understanding the initial stages of liver injury and may serve as a resource for further investigation aimed at developing new diagnostic and treatment tools.

## Results

### Identification of liver cell-type-specific transcriptional portraits under normal physiological condition

**Fig. 1:**
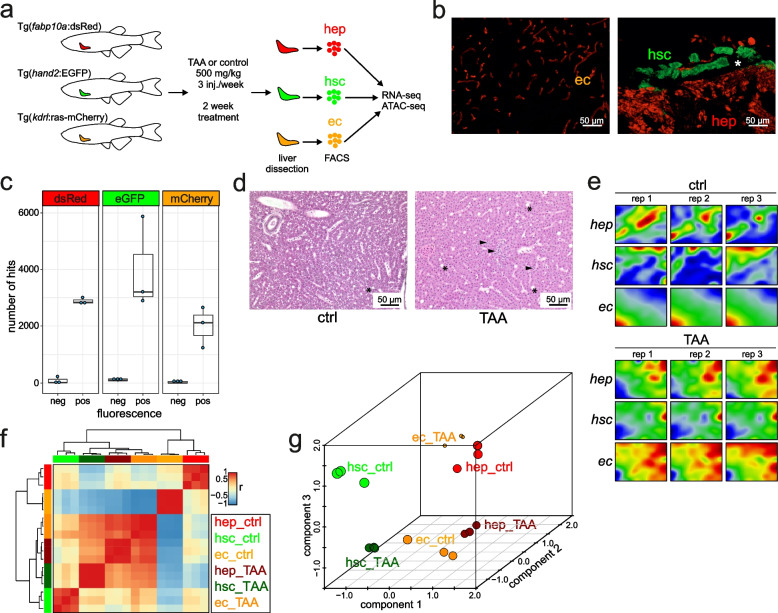
Transcriptional portraits of liver cells in response to TAA. **a** A scheme of the study. Adult transgenic zebrafish lines were treated with TAA (500 mg/kg) or control (saline) three times per week for 2 weeks. Livers were removed and fluorescent-positive cells were sorted by FACS. RNA-seq and ATAC-seq libraries were performed from sorted cells; **b** Transgenic zebrafish liver cryosection micrographs visualizing ECs (*Tg(kdrl:Hsa.HRAS-mCherry))*, HSCs (*TgBAC(hand2:EGFP))* and HEPs (*Tg(fabp10a:dsRed)*) as indicated on the figure legends; **c** Number of transgene BLAST hits from fluorescent-negative and positive cells from transgenic zebrafish lines; **d** Microscopic images of histological H&E sections of control and TAA-treated animals indicating inflammation loci (arrowheads) and extracellular lipid droplets (asterisks); **e** Portraits of co-regulated over- or underexpressed metagenes as red and blue spots, respectively. The color gradient of the map visualizes over- and underexpression of the metagenes compared with the mean expression level in the pool of all samples studied; **f** Sample pairwise Pearson correlation heatmap on the clustered data; **g** Independent Component Analysis on clustered data

**Fig. 2:**
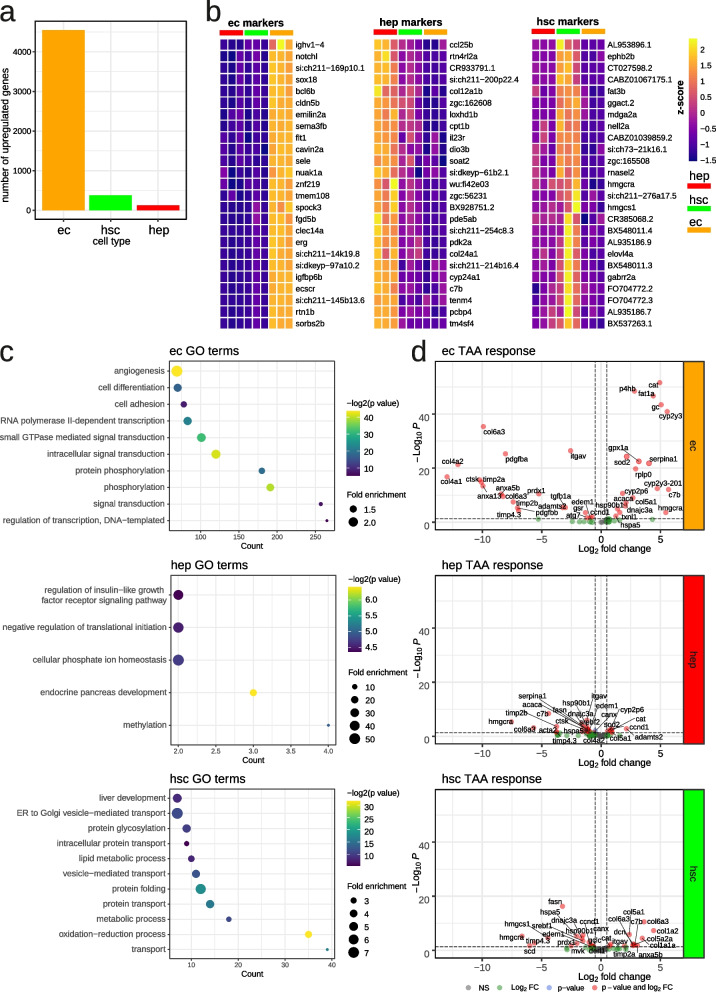
Liver cell signatures in quiescent and activated state. **a** Number of identified cell type specific genes at quiescent state in each cell type, logFC > 0, padj < 0.05; **b** Heatmaps of top 25 cell type specific genes at quiescent state in each cell type, logFC > 0, padj < 0.05; **c** GO over-representation analysis of identified cell type specific genes at quiescent state in each cell type; **d** Volcano plot of selected genes, involved in liver fibrosis and response to oxidative stress, under TAA treatment

In order to characterize the molecular profiles representing the HEPs, HSCs, and ECs under physiological conditions, we utilized three transgenic lines *Tg(fabp10a:dsRed)*, *Tg(hand2:EGFP)* and *Tg(kdrl:Hsa.HRAS-mCherry)* which express red (dsRed, mCherry) or green fluorescent proteins (GFP) in the corresponding cell types [14, 22, 23]. Whole livers were dissected from adult zebrafish from each of the transgenic lines used in this study (Fig. 1A). Fluorescent microscopy of liver from the corresponding transgenic lines confirmed the fluorescence observed in the corresponding cell types (Fig. 1B). We prepared cell suspensions and performed FACS according to previously established protocols (See Methods, Supp. Fig. 1). The number of RNA-seq reads corresponding to fluorescent reporters specific to each cell-type (Fig. 1B) was strongly enriched in fluorescent-positive samples, which confirmed the purity of FACS isolated samples (Fig. 1C). In order to ascertain the cell-type gene signatures, we performed differential expression comparisons between samples and identified the most enriched genes in each cell type (Fig. 2A, Supp. Table 2). The largest number of cell-specific genes were found in ECs (4553), then in HSCs (380) and in HEPs (126) (Supp. Table 2). These included known cell-specific markers for ECs (*sox18* [24]*, sele* [25]*, flt1* [26]*)* and HEPs (*soat2* [27]) (Fig. 2B). On the other hand, genes related to fatty acid metabolism (*fasn* [28], *fat3b, hmgcra* [29]*, hmgcs1* [30]*, elovl4a* [31]) and cholesterol biosynthesis (*cyp51, sc5d, hmgcra, msmo1, nsdhl, hmgcs1, dhcr7*) were upregulated in HSCs which are known to contain vitamin A lipid droplets [32] (Supplementary Table 2). Gene ontology (GO) analysis revealed the enrichment of genes related to angiogenesis in ECs, insulin-like growth factor receptor signalling genes and cellular phosphate ion homeostasis in HEPs and lipid transport and metabolism genes in HSCs (Fig. 2C). Taken together, the enrichment of known markers and the relevant GO terms in ECs, HEPs, and HSCs support the identity of the respective cell types.

### TAA metabolism is reflected in the transcriptional shift in liver cells

We then sought to determine the transcriptional signatures of early hepatotoxic injury response in each of the three liver cell types. We induced liver injury using TAA at a concentration of 500 mg/kg of body mass. The short term TAA treatment induced mild histological changes with observed inflammation (Fig. 1D). We then collected whole livers from TAA-treated *Tg(fabp10a:dsRed)*, *TgBAC(hand2:EGFP)* and *Tg(kdrl:Hsa.HRAS-mCherry)* fishes, isolated the corresponding cell types by FACS, and performed RNA-seq.

We evaluated cell-type-specific transcriptional response to TAA activation by looking at the expression of genes related to TAA metabolism and genes activated in response to liver injury and fibrogenesis (Fig. 2D, Supp. Table 3). The increased expression of genes related to cell redox homeostasis such as catalase (*cat*) [33], cytochromes (*cyp2y3, cyp2p6*) [34], superoxide dismutase 2 (*sod2*) [34], glutathione peroxidase 1a (*gpx1a*) [35] was observed in response to TAA, with the most striking response in ECs. Pro-fibrotic genes [8] including ECM proteins such as collagens (*col1a1a, col1a2, col5a2a, col5a1, col6a3*), decorin (*dcn*) as well as metallopeptidase inhibitor 2a (*timp2a*), integrin alpha V (*itgav*) and annexin 5b (*anxa5b*) were specifically upregulated in HSCs, in response to TAA (Fig. 2D).

### TAA induces transcriptional reprogramming of hepatic endothelial cells

**Fig. 3:**
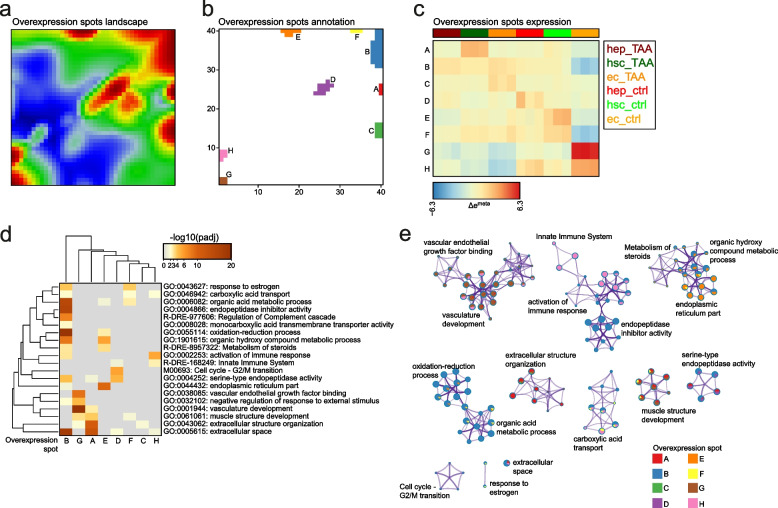
Functional characterization of overexpression spots landscape. **a** Overexpression spots landscape. Logged expression values of each gene were transformed into differential expression values relative to the mean expression of the particular gene in the experimental series of samples considered. Overexpression spots are coloured in red; **b** Overexpression spots annotation to clusters from A to H; **c** Mean overexpression spots expression across samples; **d** Gene sets enrichment analysis on the clustered data. Overrepresentation *p*-values for each cluster are provided; **e** Meta-analysis of gene set enrichment performed by Metascape. Only significantly enriched terms are shown (padj < 0.05)

To provide a global view of the behaviour of correlated gene clusters in three hepatic cell types in response to TAA, we used self-organizing map based tool oposSOM R package [36]. The tool first constructed transcriptional portraits of all the samples, then a second unsupervised reduction step was performed, further reducing dimensionality to overexpression spots representing clusters (A-H, Supp. Table 4) of co-expressed metagenes which are highly expressed in, at minimum, one condition (Fig. 3A, B) [37]. To link overexpression with gene set overrepresentation in a sample- and spot-specific way, we visualized the metagene expression across samples on the heatmap (Fig. 3C) and performed the gene set overrepresentation analysis (Fig. 3D, E; Supp. Table 5). The gene expression portraits of both control and TAA-treated samples from each of the three cell types revealed that short-term TAA exposure induced strong changes in genome-wide expression landscapes between cell types in physiological state and upon TAA activation (Fig. 1E, F). Interestingly, the most striking changes induced by TAA treatment were observed in ECs (Fig. 1G).

Analysis of the SOM clusters in ECs revealed an increase in expression of genes related to metabolic and redox processes as well as cellular transport (Fig. 3C, D - clusters B and F). We also observed downregulation of genes related to vasculature development as well as activation of immune response in ECs after treatment with TAA (Fig. 3C, D - clusters G and H; Supp. Fig. 6).

In HEPs, TAA treatment induced an increase in the expression of gene sets associated with regulation of metabolic processes, namely carboxylic acid and hydroxy compound metabolism, as well as intra- and intercellular transport when compared to their control counterparts (Fig. 3C, D - cluster B). In contrast, we observed a decreased expression of gene sets associated with the formation and function of endoplasmic reticulum as well as negative regulation of various growth binding factors (Fig. 3C, D - clusters E and G). We also observed a relative reduction of expression of genes associated with the G2/M cell cycle transition in TAA-treated HEPs (Fig. 3C, D - cluster D; Supp. Fig. 5).

Modest changes in gene expression were observed in HSCs. Analysis of clusters revealed that upregulated gene sets were associated with extracellular space and structure organization as well as protein hydrolysis (Fig. 3C, D - cluster A), which reflects the known role of HSCs in ECM formation during liver damage response [9]. Conversely, we observed downregulation of genes associated with G2/M cell cycle transition, endoplasmic reticulum, estrogen response and immune activation (Fig. 3C, D - clusters G and H).

Altogether, cell-type-specific transcriptome profile revealed transcriptional response to short term TAA exposure. All of the analyzed cell types were subject to TAA-induced transcriptional shifts, with the highest change observed in ECs. These were hallmarked by decrease of vascular-specific genes and the increase of fatty acid and carbohydrate metabolism genes as well as in immune response-associated genes.

### TAA leads to genome-wide changes in chromatin regions enriched in binding sites for transcription factors regulating fatty acid metabolism and angiogenesis

Epigenetics has been acknowledged as an important player in liver fibrosis and regeneration [38–40], with a prospect of the development of epigenetic biomarkers and therapies. To investigate this aspect of liver damage, we ask whether epigenetic changes are involved in the earliest stages of liver fibrosis. To determine whether and to what extent epigenetic landscape in each liver cell type is altered during early stage liver injury, we characterized the changes in chromatin accessibility in HEPs, HSCs, and ECs upon TAA treatment.

**Fig. 4:**
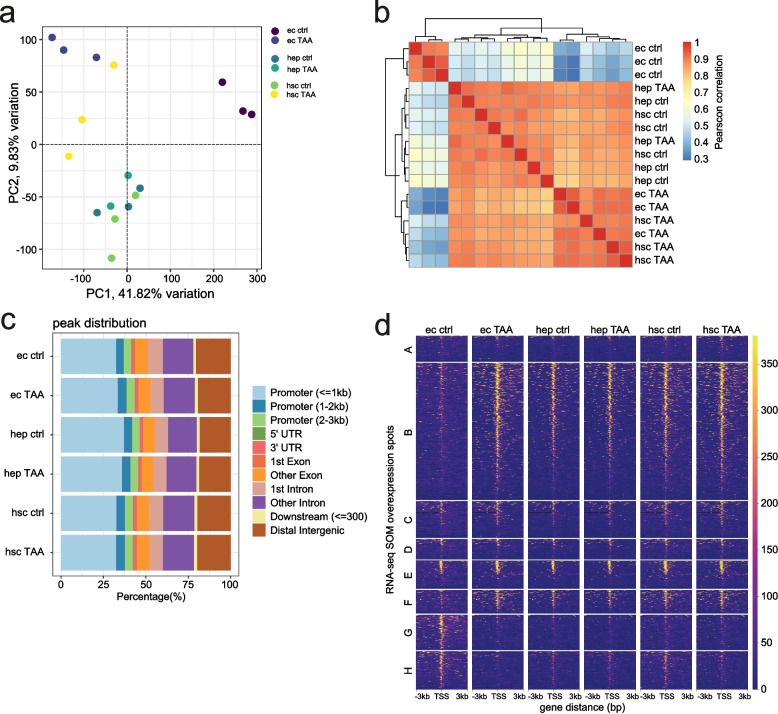
Chromatin accessibility maps of liver cells. **a** Principal component analysis of ATAC-seq peaks across cell types and conditions; **b** Sample pairwise Pearson correlation heatmap of chromatin accessibility in ATAC-seq peaks across cell types and conditions; **c** ATAC-seq peak distribution across genomic categories; **d** Coverage heatmaps of ATAC-seq peaks localized in the promoters (− 3 to + 3 kb from TSS) of SOM clusters

**Fig. 5:**
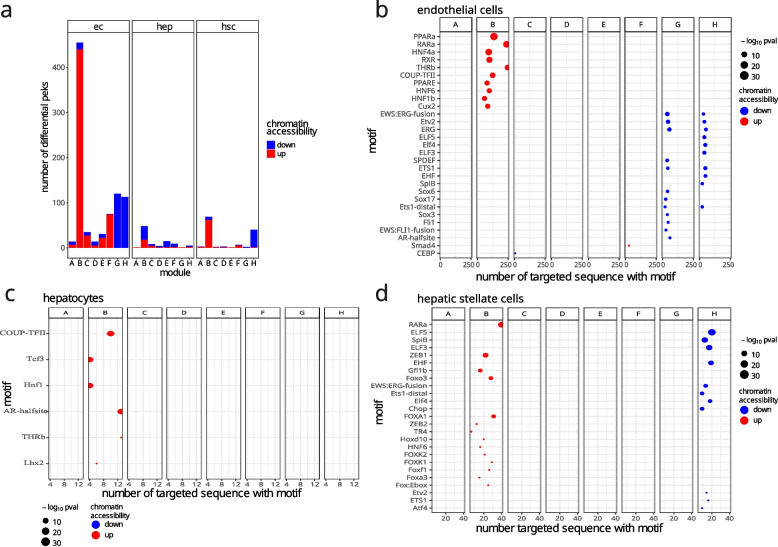
TF motif enrichment in response to pro-fibrotic stimuli. **a** Metrics of differential promoter peaks (− 3 to + 3 kb from TSS) in SOM clusters; **b** Homer motif enrichment analysis in ECs differential peaks; **c** Homer motif enrichment analysis in HEPs differential peaks; **d** Homer motif enrichment analysis in HSCs differential peaks. Only enriched motifs with p-adjusted < 0.1 are shown

We observed that in TAA-treated animals the most significant changes in chromatin state compared to control were observed in ECs, followed by HSCs and HEPs (Fig. 4A, B). ATAC-seq peaks distribution across the genome showed that the highest fraction of peaks (30-40%) was localized in the promoter (+/− 3 kb) regions (Fig. 4C, Supp. Table 7). Interestingly, the most significant changes in chromatin accessibility was observed in ECs, with the largest number of upregulated peaks found within the promoters of genes in clusters B (440 peaks) and F (74 peaks) and downregulated peaks in clusters G (120 peaks) and H (113 peaks) (Fig. 5A). The observed changes in chromatin accessibility correlates with changes observed in the transcriptional levels of genes within the corresponding clusters (increase in clusters B and F, and decrease in clusters G and H) (Fig. 4D). On the other hand, modest changes in chromatin accessibility were observed in the other two cell types. In HEPs, the highest change was observed in cluster B (30 up- and 18 downregulated). In HSC, 62 and 7 peaks were upregulated or downregulated in cluster B, respectively and 39 downregulated in cluster H.

To identify potential regulators involved in TAA response in each cell type, we searched for transcription factor (TF) motifs enriched in differentially accessible promoter peaks from SOM cluster genes (Fig. 5B-D, Supp. Table 6). Significant enrichments (p-adjusted < 0.05, Supp. Table 6) were identified predominantly in five tested groups of regions: cluster B upregulated regions in ECs and HSCs, cluster G downregulated regions in ECs and cluster H downregulated regions in ECs and HSCs. In ECs, we observed significant enrichment in motifs of fatty acid metabolism nuclear receptors such as RXR [41], THRB [42], HNF4A [43] and PPARA [41] among peaks upregulated in cluster B. This is in accordance with the result of gene set overrepresentation analysis (Fig. 3D). A drop in chromatin accessibility was observed for ECs peaks located in the promoter of genes from cluster G. TFs motifs identified in this cluster belong to ETS family (ETV2, ERG, SPDEF, ETS1) and Sox family (Sox6, Sox17, Sox3) involved in cell differentiation, migration and proliferation [44–46]. In HSCs, we found enriched motifs of TFs involved in cellular glucose homeostasis such as FOXA3 [47], FOXK1 [48], FOXK2 [49] and cell differentiation such as RARA, TR4, FOXA1, FOXA3 [50]. In cluster H downregulated regions, both in EC and HSC, we also found enriched motifs of ETS family including ETV2, ERG, ELF5, ELF3, ETS1, EHF, SPIB, ELF4. Additionally, in HSCs we found enrichment of ATF4 and Chop motifs, which are known to be involved in response to endoplasmic reticulum stress [51, 52]. Notably, ETS TFs also regulate endothelial function and homeostasis [53]. Altogether, our results show increased chromatin accessibility in the promoter regions of gene clusters associated with fatty acid metabolism, especially in ECs, and decrease of accessibility in clusters related to endothelial homeostasis and inflammatory response.

### ECs exhibit the highest gene regulatory response to TAA-induced liver injury

**Fig. 6:**
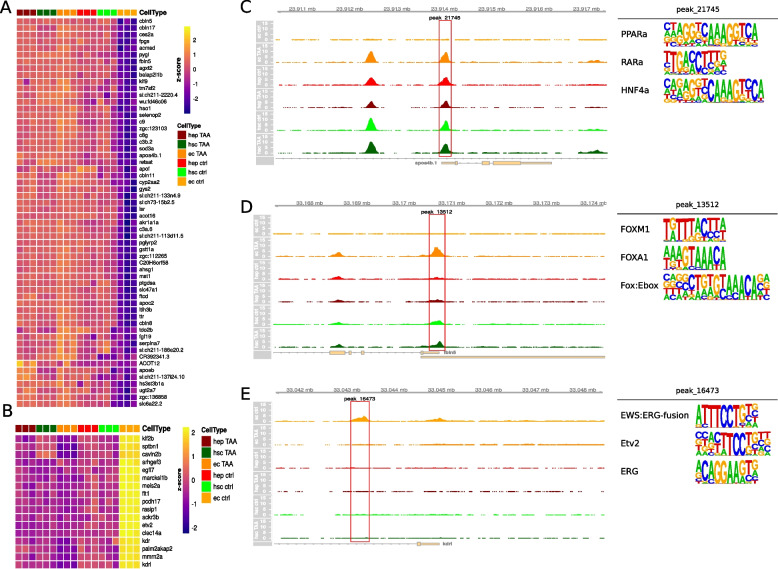
Cell type specific accessibility changes in response to TAA treatment in selected cell types and clusters. **a** Heatmap of selected genes in each cell type. Genes were selected based on accessibility patterns in cluster B; **b** Heatmap of selected genes in each cell type. Genes were selected based on accessibility patterns in cluster B; **c** Genomic browser snapshot at *apoa4b.1* promoter localization with accessibility track expressed as reads per million. Highlighted peak was used as a selection criteria in **a**., its three most enriched motifs are shown next to the browser track; **d** Genomic browser snapshot at *fbln5* promoter localization with accessibility track expressed as reads per million. Highlighted peak was used as a selection criteria in **a**., its three most enriched motifs are shown next to the browser track; **e** Genomic browser snapshot at *kdrl* promoter localization with accessibility track expressed as reads per million. Highlighted peak was used as a selection criteria in **b**., its three most enriched motifs are shown next to the browser track

To further investigate cell type specific responses to TAA treatment we examined the character of promoter accessibility change in clusters most specific to each cell type. These included clusters B, G and H in ECs and cluster A in HSCs. In cluster B we observe the tendency in ECs towards increase in promoter accessibility upon treatment (Fig. 4D and Supp. Fig. 3B) combined with increase in expression (Fig. 3C). Among the genes that increase in accessibility, we focused on those that exemplify the largest gain in accessibility by selecting the top 25th percentile of change in accessibility and lower 25th percentile of read counts in the control sample (Fig. 6A). Among those were homologs of known human liver fibrosis markers such as Apolipoprotein A-IV [54] or Fibulin-5 [55] (Fig. 6C, D). In clusters G and H we observe a decrease in promoter accessibility (Fig. 4D, Supp. Fig. 3C, D) accompanied by reduced gene expression (Fig. 3C). To select genes with the most prominent loss of accessible regions in their promoter after treatment, we examined differentially accessible regions in the lower 25th percentile in terms of accessibility change and upper 25th percentile in read counts in the control sample (Fig. 6B, Supp. Fig. 4A). Among such genes in cluster G were EC marker *kdrl* and known vascular endothelial regulator *etv2* [56] (Fig. 6E, Supp. Fig. 4C). In contrast, a limited number of changes were observed in promoter accessibility of HSCs in cluster A (Fig. 4D and Supp. Fig. 3A). Among the 5 genes within the top 25th percentile of accessibility changes and lower 25th percentile of read counts in control were *col4a6* and *elovl1a* (Supp. Fig. 4B, D, E).

## Discussion

Liver fibrosis is a wound-healing response to tissue injury and inflammation hallmarked by the ECM protein deposition in the liver parenchyma and tissue remodelling [57]. The predominant causes of liver fibrosis are chronic excessive alcohol consumption, viral hepatitis B and C and non-alcoholic fatty liver disease (NAFLD), the latter becoming a major concern with the increasing incidence of obesity in Europe and the USA [1]. While these conditions have been widely studied [1], current knowledge of gene regulatory networks and epigenetic hallmarks associated with the early response to hepatotoxic injury is still lacking. It is crucial to study these primary changes in the cell types most affected by injury to improve the tools for diagnosis of early liver fibrosis and related disorders. In order to dissect the molecular mechanisms regulating the initiation of hepatic fibrosis and understand the interplay between genetic and epigenetic signals in this process, we utilized the model of TAA-induced liver injury in adult zebrafish and characterized cell-type-specific changes at both transcriptome and epigenome level in three main liver cell types: HEPs, HSCs and ECs.

The conservation of many metabolic pathways across vertebrate species renders the zebrafish a potent model organism in drug discovery studies. It has been extensively used to study liver development and injury [58, 59], and has been especially useful in establishing various toxicity models [60]. Many xenobiotics used to establish murine models of drug-induced liver injury have been found to be as effective in zebrafish, with an added advantage of the larvae being suitable for toxicological studies at 3 days post-fertilization, when mature liver parenchyma can be observed [60]. While the zebrafish liver architecture is distinct from its mammalian counterpart, the morphology, localization and gene expression profiles of HEPs, ECs and HSCs are similar [58, 60, 61].

The hepatotoxic properties of TAA in mice and rats induces oxidative stress resulting first in formation of intracellular lipid deposits in the liver parenchymal cells (hepatocyte ballooning), and later leading to HEPs damage and necrosis [62]. Bioactivation of TAA into its hepatotoxic counterpart, TASO_2_ [63], requires proteins from the cytochrome p450 complex, functional orthologs for many of which exist in zebrafish, including proteins with > 44.87% sequence similarity to CYP2E1, the protein thought to be directly responsible for TAA metabolism in humans [64]. Moreover, CYP2E1 function was reproduced in zebrafish tissue homogenates, albeit without identifying the specific enzyme responsible for the process [65].

In line with previous reports [5, 66], we observed that gene expression profiles of respective cell populations are similar to those exhibited by their mammalian counterparts. Specifically, our sorted cell populations were enriched for known cell specific markers and relevant GO terms. These results are in agreement with the established existence of conserved molecular pathways between species [58]. Moreover, our analysis of cell-type-specific transcriptional response to TAA treatment highlighted known molecular components of the TAA metabolism pathway such as elements of the cytochrome p450 superfamily (Supp. Table 3). The most striking transcriptional response to TAA was observed in the ECs, highlighting those cells as the most affected by the treatment. This is likely a consequence of high permeability of ECs and also reflects their driving role in hepatotoxic injury response [67]. ECs, particularly LSEC, due to their exceptional permeability and intimate contact with the blood stream [68], are at the frontline of the toxic stimuli sensing. During liver damage, endothelial dysfunction occurs at early phases, before fibrosis initiation [69–71], under many liver etiologies such as non-alcoholic fatty liver disease (NAFLD) and alcoholic liver damage. Some evidence shows that LSEC dysfunction occurs before other liver injury early markers including Kupffer cell activation, nitric oxide content reduction or TNFα, IL-6 and ICAM-1 up-regulation [67, 70, 72]. To accompany their high toxins susceptibility ECs play a regulatory role in the liver cellular response to an injuring factor [67]. The main target of this regulation are the hepatic stellate cells (HSC), but evidence was shown on ECs involvement in control of HEPs proliferation [73]. In chronic models of liver injury, ECs, specifically LSEC, can generate a strong immune response and became highly proinflammatory, while secreting a vast range of cytokines and chemokines including TNF-α, IL-6, IL-1, CCL2 [67]. In response to those stimuli as well as the damaging toxin, other cells co-participate in the liver cellular response regulation. Injured hepatocytes and inflammatory cells secrete inflammatory mediators, which further stimulate LSEC and the inflammatory response.

To assess TAA-induced transcriptional changes in more detail, we applied SOM to identify clusters of co-expressed genes in our transcriptome data. We found eight clusters that showed greatest variability between conditions. The largest of these, cluster B, showed highest upregulation in response to TAA treatment in ECs. Interestingly, this cluster consists of genes related to metabolic and redox processes, including 20 members of the cytochrome p450 superfamily. This suggests that cluster B represents the set of genes most directly responding to TAA treatment. The expression of CYP2E1 in LSECs was recently reported in the case of alcohol induced liver injury in mice [74]. Moreover, in agreement with the ability of ECs to regulate neighboring cells, eg. via angiocrine factors, we found many genes whose products are known to localize in the extracellular space in cluster B. This includes Apolipoprotein A-IV which has been recently identified as a potent liver fibrosis biomarker [54]. Conversely, clusters G and H showed strong downregulation upon TAA treatment. Of these, genes involved in extracellular structure organisation (cluster G) showed the strongest response in the ECs, while genes involved in immune response (cluster H) were commonly downregulated across all cell types. Contrary to previous reports [75, 76], we did not observe an upregulation of extracellular space-associated genes, especially matrix metalloproteinase genes (clusters A and C) in HEPs. This may be due to the differences in experimental design, as in contrast to the cited studies we investigated the earliest stages of liver injury. Other possible sources of divergent results may be the choice of hepatotoxin, as both cited studies employed CCl_4_. This result could also highlight the differences in model organisms of choice, as the cited studies have employed mice, rats and human cell lines.

The observed gene expression upregulation in response to treatment is accompanied by increased promoter accessibility. In agreement with RNA-seq data, we observe the largest chromatin rearrangements in ECs. This result suggests that chromatin remodeling is an important mechanism driving gene expression response to liver injury. Indeed, our motif enrichment analysis identified known motifs of transcriptional activators, such as the pioneer factors *foxa1* and *foxa3*, to be enriched in the regions of increased accessibility. Curiously, the murine homolog of *foxa3* has been implicated in promoting liver regeneration [77], while *foxa1* is important for proper liver parenchyma development [78]. Changes in promoter accessibility in other cell types were less prominent, however the increase in chromatin accessibility was observed in HSCs’ *col4a6* promoter region upon TAA treatment. This, taken together with the increased transcription of ECM genes in both ECs and HSCs can suggest that the initiation of ECM remodeling driven by both these cell types is triggered by hepatic injury.

## Conclusions

We induced liver injury using TAA, an established potent hepatotoxin, in adult zebrafish. Using this system, we identified cell-type specific response to early hepatotoxic liver injury at the transcriptomic and regulatory level. We demonstrated that in zebrafish, the first major liver cell population exposed to hepatotoxin - ECs - is also the most affected at both transcriptomic and chromatin accessibility level at this stage of liver injury. Importantly, genes known to be key players in ECM remodelling as well as metabolic and redox processes were observed to be responsive to TAA-mediated liver injury, including some which undergo chromatin re-arrangement at their promoter regions. Besides revealing the global transcriptome and epigenome landscape of early liver injury, this work provides insight into the molecular processes involved in early stages of liver damage. It also promises the viability of employing approaches providing even more specific, in-depth information, such as single cell sequencing or long read sequencing. These could potentially allow researchers to identify subpopulations of cells within major cell types that are responsible for distinct signals and injury response patterns, or assess transcript modifications triggered by early liver injury.

## Methods

### TAA dose-response assessment

Treatment of adult zebrafish individuals with TAA at a concentration of 300 mg/kg b.m. which was previously reported for female zebrafish [20] did not result in morphological changes compared to saline-injected controls (Supp. Fig. 2), thus suggesting that a higher concentration of TAA is required to induce liver injury in adult fish. In order to establish the optimal TAA concentration for adult zebrafish, we first performed a range-finding experiment to identify the working dose for zebrafish embryos, which we would then use as a guideline for establishing the higher dose in adults. By performing the toxicity assay in embryos instead of adults we bypassed the need to sacrifice large numbers of animals. Embryos at 48 hpf (*n* = 18 for each concentration) were placed individually in 12-well plates. 5 concentrations were tested: 150 mg/l, 375 mg/l, 750 mg/l, 1500 mg/l and 3750 mg/l. The TAA solution was changed every 24 h for 72 h, at which point the embryo survival was estimated. A control group for each concentration was kept in E3 medium (5 mM NaCl, 0.17 mM KCl, 0.33 mM CaCl_2_, 0.33 mM MgSO_4_) and changed every 24 h for the duration of the experiment. We found that treatment of embryos with 1500 mg/l of TAA for 72 h resulted in ~ 50% mortality, thereby approximating the embryonic LC50 for TAA at this concentration. To ensure an adequate amount of TAA delivered to the adult liver, we adopted the intraperitoneal injection strategy repeated 6 times over the span of 2 weeks, with a dose of 500 mg/kg of body mass per injection.

### TAA administration and isolation of liver cell populations by fluorescence-activated cell sorting (FACS)

Zebrafish transgenic lines *Tg(fabp10a:dsRed)*, *Tg(hand2:EGFP)* and *Tg(kdrl:ras-mCherry)* in AB wild-type background were maintained in the IIMCB zebrafish facility (License no. PL14656251) according to standard procedures. Adult females were anesthetized with MS-222 (Sigma-Aldrich, Germany) as previously described [79] and injected intraperitoneally with 500 mg/kg thioacetamide (TAA) or sterile water as a control 6 times over the course of 2 weeks. A single dose of TAA would not approach the estimated LC50 for embryos, but the overall exposure to the toxin would exceed the estimated LC50. Adult fish weighing less than 2 g prior to the injections were excluded due to welfare concerns. Prior to toxin administration, the injection spot was wiped down with 1% povidone iodine to further limit the risk of infection. Overall, 15 fishes were injected with TAA. An additional 6 were injected with saline as a control. Fishes injected with TAA survived to the end of the 2-week treatment with 20% mortality (n surviving = 12). All saline-injected fishes survived the procedure. Experimental protocol for the treatment of animals in this study follows the guidelines approved by First Warsaw Local Ethics Committee for Animal Experimentation (file 15/2015). Livers were dissected and digested in Hank’s solution (1× HBSS, 2 mg/mL BSA, 10 mM Hepes pH 8.0) containing 0.05% trypsin (Sigma-Aldrich, Germany) and 2% collagenase (Sigma-Aldrich, Germany). Cell suspension was centrifuged at 500 g for 10 min at 4 °C. Cell pellet was resuspended in FACSmax (Amsbio, UK) and passed through a sterile 0.22 μm cell strainer (VWR, USA). Fluorescent cells were sorted by using FACSAria II cytometer (BD Biosciences, USA).

### RNA-seq

For RNA sequencing 100,000 fluorescent liver cells were sorted directly to TRIzol LS (Thermo Fisher Scientific, USA). After ethanol precipitation RNA was depleted of DNA by using DNase I treatment and purified on columns by using RNA Clean & Concentrator™-5 (Zymo Research, USA). RNA integrity was measured by RNA ScreenTape on the Agilent 2200 TapeStation system (Agilent Technologies, USA). RNA Integrity Number (RIN) was in the range from 8.5 to 10 for all the samples used for RNA-seq. Ribosomal RNA removal from 10 ng of total RNA was performed using RiboGone Kit (Clontech Laboratories, USA). cDNA synthesis for next-generation sequencing (NGS) was performed by SMARTer Universal Low Input RNA Kit (Clontech Laboratories, USA) as recommended by the manufacturer. DNA libraries were purified with Agencourt AMPure XP PCR purification beads (Beckman Coulter, USA) and DNA fragment distribution was assessed by using D1000 ScreenTape and Agilent 2200 TapeStation system (Agilent Technologies, USA). KAPA library quantification kit (Kapa Biosystems, USA) was used for qPCR-based quantification of the libraries obtained. Paired-end sequencing (2 × 75 bp reads) was performed with NextSeq 500 sequencing system (Illumina, USA).

### ATAC-seq

For ATAC-seq 60,000 fluorescent liver cells were sorted to Hank’s solution (1× HBSS, 2 mg/mL BSA, 10 mM Hepes pH 8.0), centrifuged for 5 min at 500×g and prepared for chromatin tagmentation as previously described [80]. NEBNext High-Fidelity 2 × PCR Master Mix (New England Biolabs, USA) and custom HPLC-purified primers containing Illumina-compatible indexes were used to prepare DNA sequencing libraries as previously described [81]. DNA libraries were purified with Agencourt AMPure XP PCR purification beads (Beckman Coulter, USA) and DNA fragment distribution was assessed by using D1000 ScreenTape and Agilent 2200 TapeStation system (Agilent Technologies, USA). KAPA library quantification kit (Kapa Biosystems, USA) was used for qPCR-based quantification of the libraries obtained. Paired-end sequencing (2 × 75 bp reads) was performed with NextSeq500 sequencing system (Illumina, USA).

### Bioinformatics analysis

Raw RNA-seq and ATAC-seq reads were quality checked using Fastqc (0.11.8). Adapters were removed using Cutadapt (1.18) [82]. RNA-seq reads matching ribosomal RNA were removed using rRNAdust [83] and remaining reads were aligned to the zebrafish reference genome (GRCz11) using STAR (2.6) [84]. ATAC-seq reads were aligned to the zebrafish reference genome (GRCz11) using Bowtie2 (2.3.4.3) [85]. Reads quality filtering was performed using SAMtools (1.9) [86]. Read and alignment quality reports were prepared in Multiqc (1.6). To identify nucleosome free regions (NFRs) ATAC-seq reads originating from fragments not longer than 128 bp were retained and shifted by + 4 / -5 bp depending on the alignment strand using alignmentSieve utility from deepTools suite (3.2.0) [87]. Those reads were further used for peak calling using Macs2 (2.1.0.2) [88] subcommands. Shortly for each of the three replicates per base enrichment *p*-value track was calculated using the Poisson test. Then *p*-values tracks from replicates were combined using Fisher method. After Benjamini - Hochberg multiple testing correction, peaks were called on obtained tracks with q-value cutoff of 1e-5. Further obtained BED files were manipulated using Bedtools (2.27.1) [89] to discard NFRs overlapping low complexity regions as defined in the Ensembl’s [90] reference genome (GRCz11). Enriched motifs in NFRs were identified using Homer (4.10) [91]. Downstream bioinformatics analysis were performed in R 3.4.4 using several Bioconductor [92] packages. Cell type specific genes at quiescent state, were identified using DESeq2 [93] by comparing gene expression in specific cell type with gene expression in the other two. High-dimensional portraying of gene expression profiles was performed using oposSOM [36]. Differential gene expression analysis and differential accessibility analysis was performed using DESeq2 [93]. ATAC-seq peaks were processed and visualized using ChIPseeker [94], clusterProfiler [95], rtracklayer [96] and Gviz [97].

### Histology and fluorescent microscopy

Adult females were sacrificed by overdosing MS-222 (Sigma-Aldrich, Germany) as previously described [98]. Samples were fixed in Dietrich’s fixative [98], dehydrated in ethanol and embedded in JB-4 resin (Sigma-Aldrich, Germany) for 3 h at 4 °C. Liver histology was examined microscopically in sections (4 μm thick) after hematoxylin and eosin (Sigma-Aldrich, Germany) staining using a modified protocol with increased staining and wash times to account for the lower staining efficiency in JB-4 resin. To detect fluorescence of GFP, mCherry and RFP, livers were fixed in 4% formaldehyde, incubated overnight in 20% sucrose, frozen in OCT solution (Leica Biosystems, France) and viewed under fluorescence microscope after sectioning (section thickness = 15 μm).

## Supplementary Information

**Additional file 1.**

**Additional file 2.**

## Data Availability

RNA-seq and ATAC-seq data have been submitted to the NCBI Gene Expression Omnibus database (https://www.ncbi.nlm.nih.gov/geo/) under accession number GSE145565.

## Abbreviations

ECM: Extracellular matrix
TAA: Thioacetamide
HEP: Hepatocyte
HCC: Hepatocellular carcinoma
NAFLD: Non-alcoholic fatty liver disease
HSC: Hepatic stellate cell
LSEC: Liver sinusoidal endothelial cell
EC: Endothelial cell
SOM: Self-organising map
FACS: Fluorescence-activated cell sorting
GO: Gene ontology
TF: Transcription factor
