# Supplementary material for: Multi-omics analyses of early liver injury reveals cell-type-specific transcriptional and epigenomic shift
Source: BMC Genomics. 2021 Dec 18;22:904. doi: 10.1186/s12864-021-08173-1 (PMC8684102; doi:10.1186/s12864-021-08173-1)
Supplement: Supplementary file 1 — Additional file 1. [file 12864_2021_8173_MOESM1_ESM.pdf]

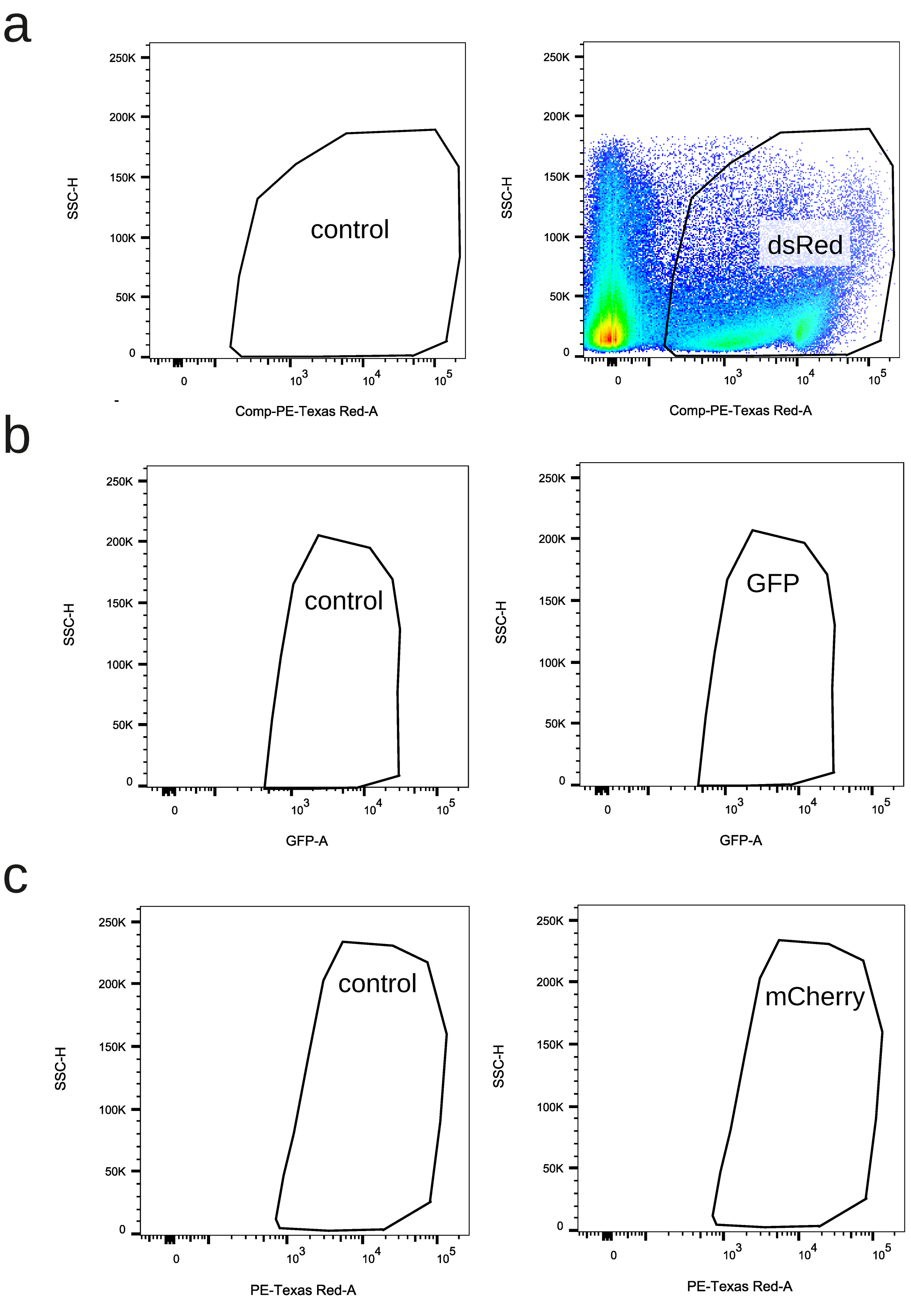

Supplementary Figure 1. FACS-sorting on liver cell populations.
SSC-A vs fluorochrome-A was applied to identify fluorescent cells.

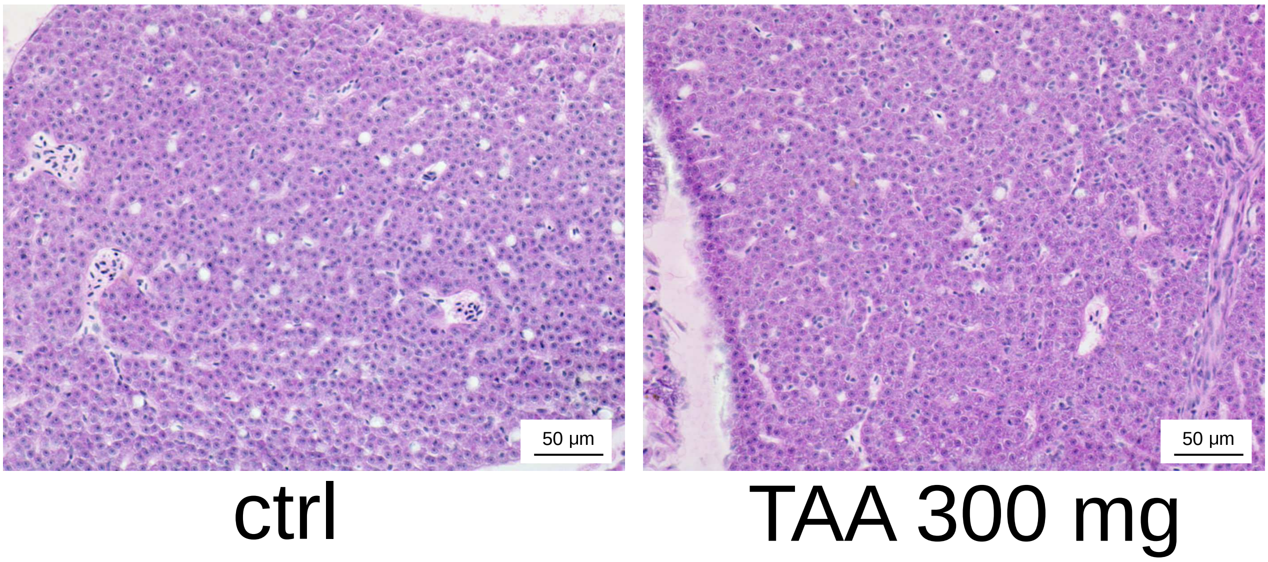

Supplementary Figure 2. Micrographs of liver sections from control and 300mg/kg b.m. TAA-treated zebrafish. Images were taken with the ZEISS Imager M1 microscope using the 20X magnification lens. Red, Green and Blue channel images were merged using ImageJ software.

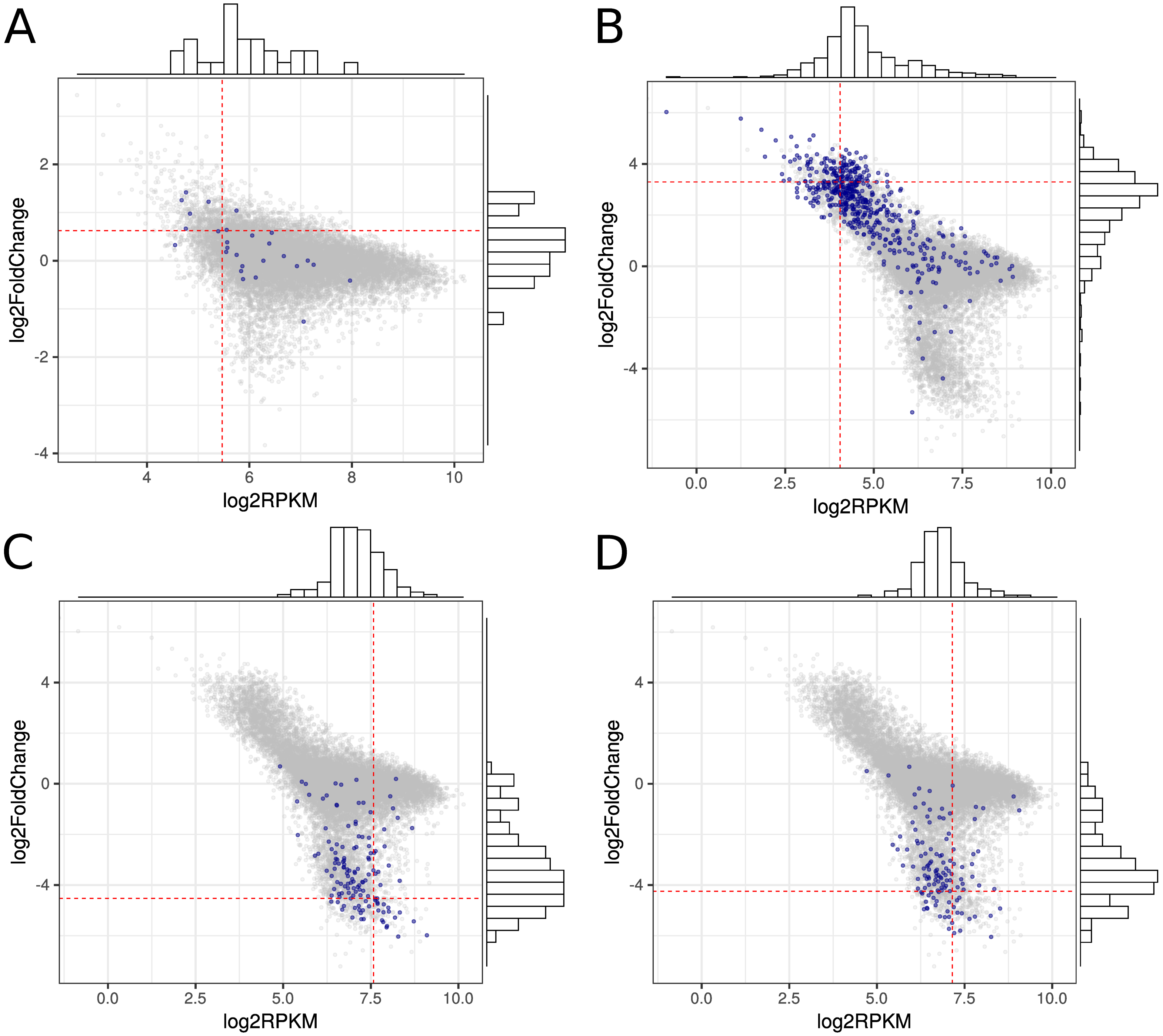

Supplementary Figure 3. Accessibility levels and change in response to treatment in selected cell types and clusters. a. Scatterplot presents the relation between accessibility level in control HSCs expressed as log2 reads per kilobase million and change in accessibility between control and treated HSCs expressed as log2 fold change. Blue points present peaks associated with genes from cluster A, grey points all peaks in HSCs. Red dotted lines mark the 25th percentile on each axis; b. Scatterplot presents the relation between accessibility level in control ECs expressed as log2 reads per kilobase million and change in accessibility between control and treated ECs expressed as log2 fold change. Blue points present peaks associated with genes from cluster B, grey points all peaks in HSCs. Red dotted lines mark the 25th percentile on each axis; c. Scatterplot presents the relation between accessibility level in control ECs expressed as log2 reads per kilobase million and change in accessibility between control and treated ECs expressed as log2 fold change. Blue points present peaks associated with genes from cluster G, grey points all peaks in HSCs. Red dotted lines mark the 25th percentile on each axis; d. Scatterplot presents the relation between accessibility level in control ECs expressed as log2 reads per kilobase million and change in accessibility between control and treated ECs expressed as log2 fold change. Blue points present peaks associated with genes from cluster H, grey points all peaks in HSCs. Red dotted lines mark the 25th percentile on each axis.

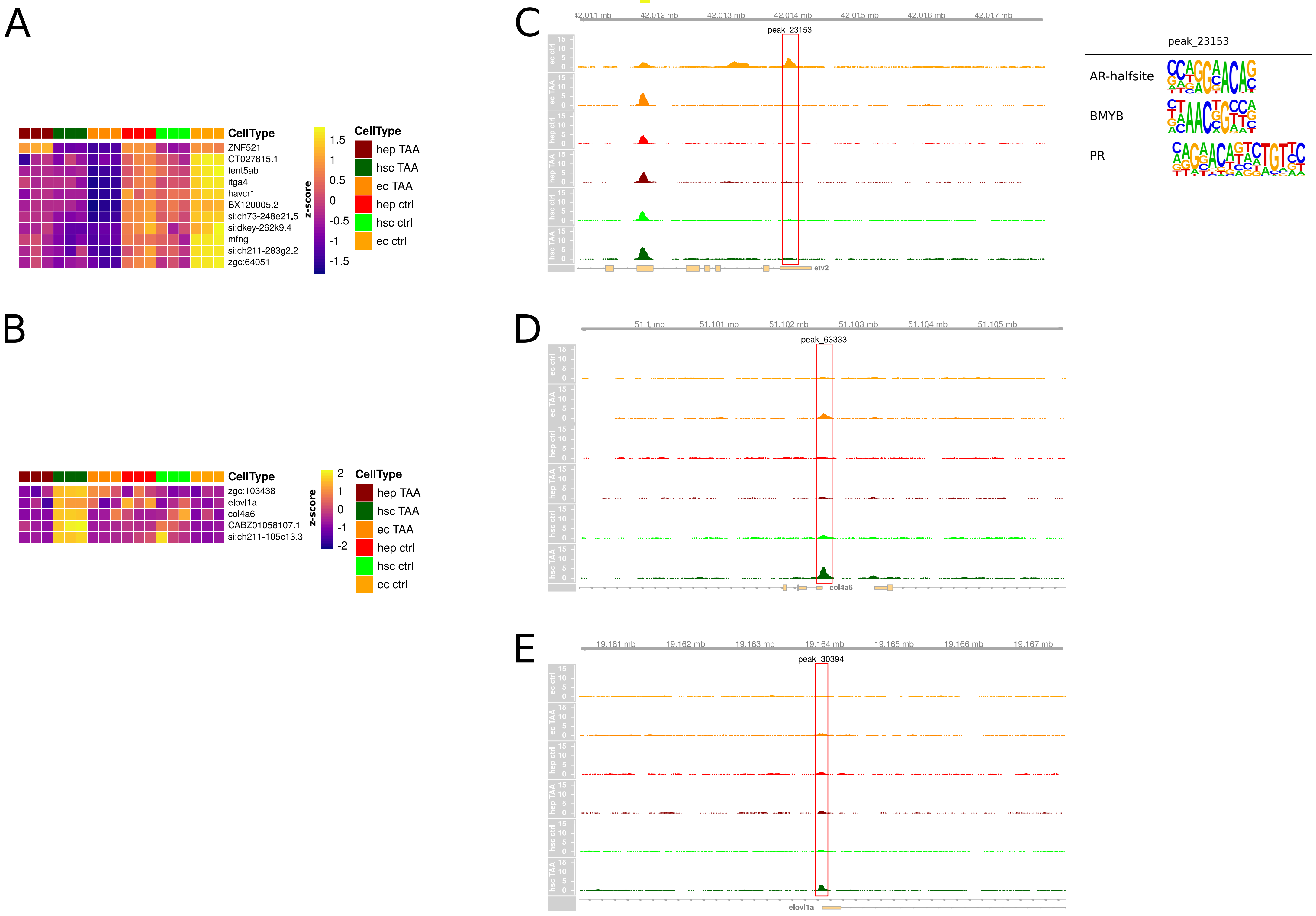

Supplementary Figure 4. Cell type specific accessibility changes in response to TAA treatment.

Supplementary Figure 4. Cell type specific accessibility changes in response to TAA treatment.

a. Heatmap of selected genes in each cell type. Genes were selected based on accessibility patterns from genes in cluster H; b. Heatmap of selected genes in each cell type. Genes were selected based on accessibility patterns from genes in cluster A; c. Genomic browser snapshot at etv2 promoter localization with accessibility track expressed as reads per million. Highlighted peak was used as a selection criteria in Fig. 6B., its three most enriched motifs are shown next to the browser track; d. Genomic browser snapshot at col4a6 promoter localization with accessibility track expressed as reads per million. Highlighted peak was used as a selection criteria in b.; e. Genomic browser snapshot at elovl1a promoter localization with accessibility track expressed as reads per million. Highlighted peak was used as a selection criteria in b.

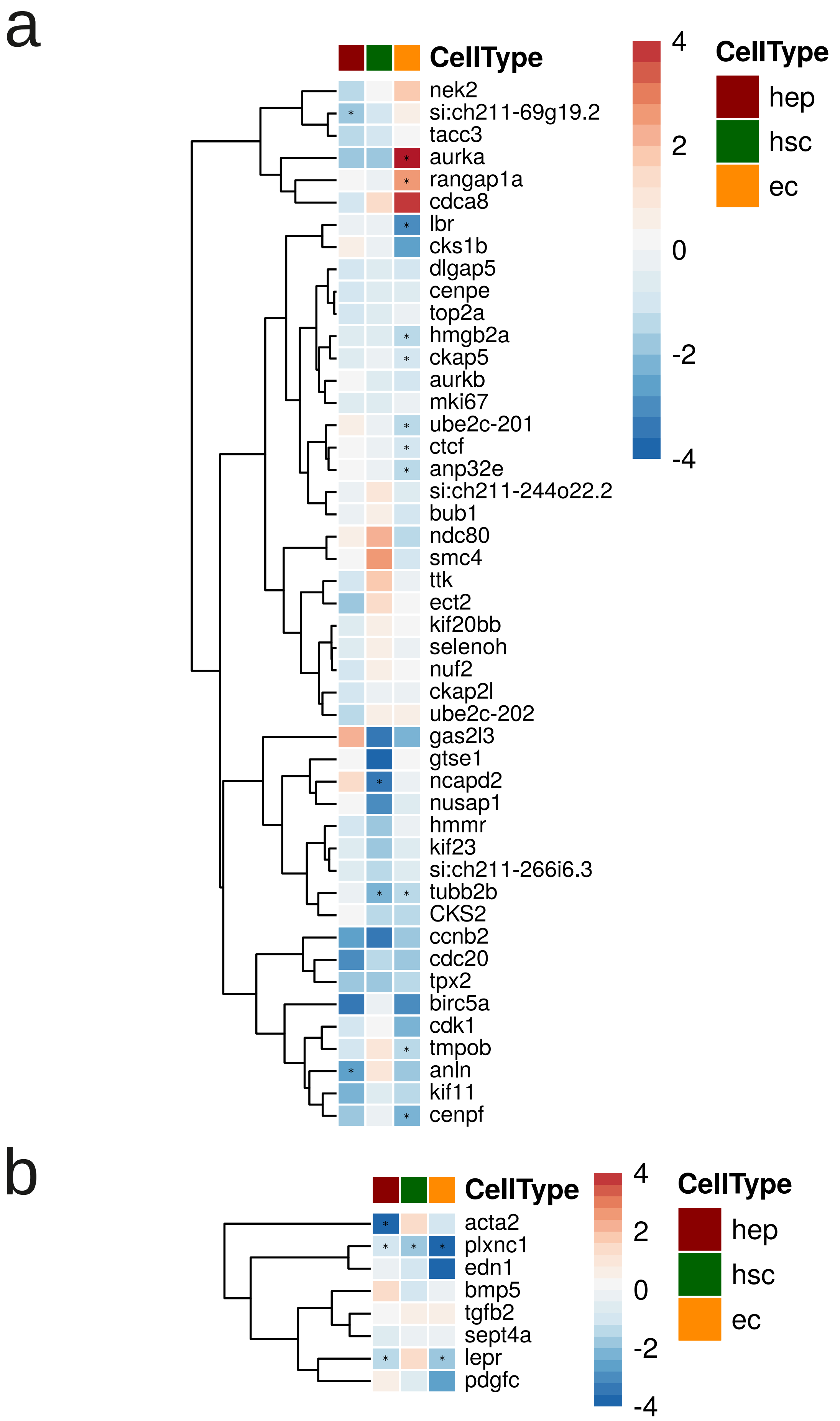

Supplementary Figure 5. TAA induced changes in expression of selected marker genes. a. Heatmap of G2M marker genes [99] log2FoldChanges after TAA treatment in corresponding cell types. Significantly differential genes were marked with an asterix. B. Heatmap of HSC activation marker genes [100] log2FoldChanges after TAA treatment in corresponding cell types. Significantly differential genes were marked with an asterix.

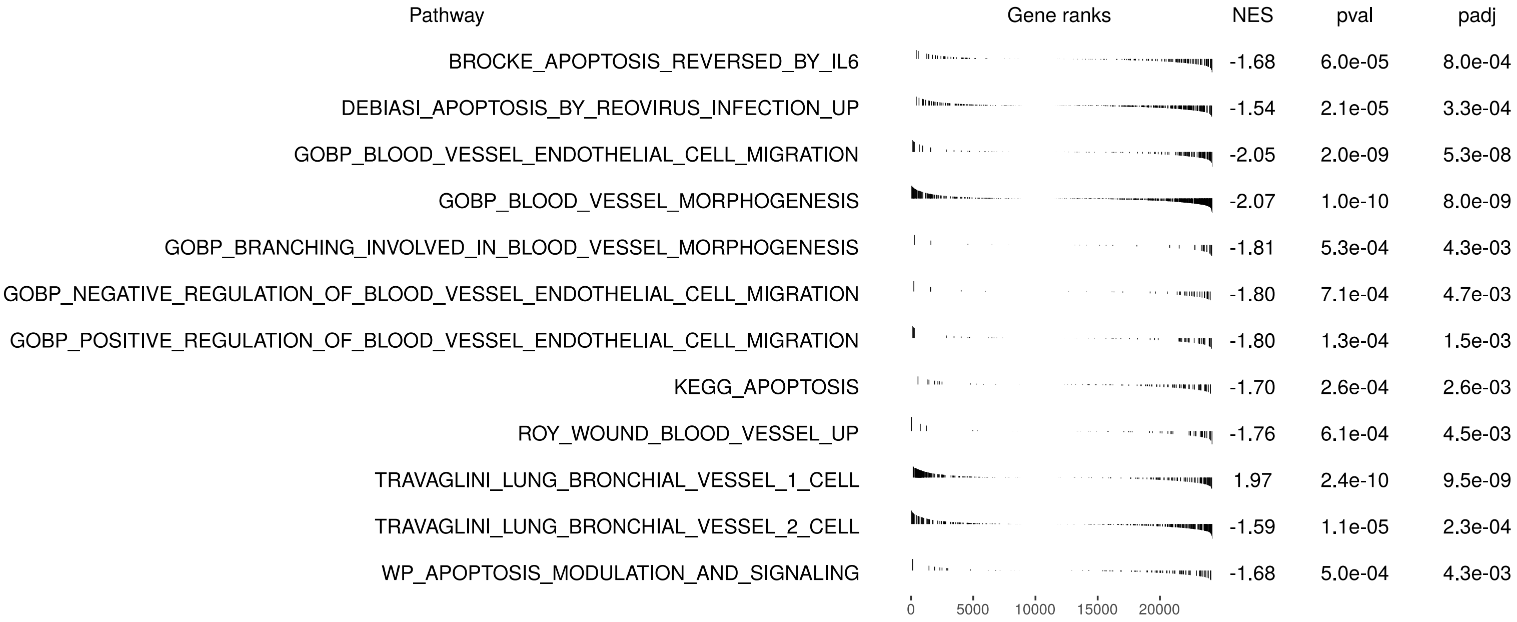

Supplementary Figure 6. Gene Set Enrichment Analysis of selected molecular signatures. Gene Set Enrichment Analysis of the apoptosis and vessel related molecular signatures in TAA induced EC.
